# Supplementary material for: Alkaloid Derivative (Z)-3β-Ethylamino-Pregn-17(20)-en Inhibits Triple-Negative Breast Cancer Metastasis and Angiogenesis by Targeting HSP90α
Source: Molecules. 2022 Oct 21;27(20):7132. doi: 10.3390/molecules27207132 (PMC9611734; doi:10.3390/molecules27207132)
Supplement: Supplementary file 1 [file molecules-27-07132-s001.zip › molecules-1964436-supplementary.pdf]

Supplementary Materials

Alkaloid Derivative (Z)-3β-Ethylamino-pregn-17(20)-en Inhibits Triple-Negative Breast Cancer Metastasis and Angiogenesis By Targeting HSP90α

Xin-Yao Liu[1,†],Yu-Miao Wang[1,†], Xiang-Yu Zhang[1], Mei-Qi jia[1], Hong-Quan Duan[1,2,3], Nan Qin[1], Ying Chen[1], Yang Yu[1*], Xiao-Chuan Duan[1,4*]

[1] School of Pharmacy, Tianjin Medical University, Tianjin 300070, China

[2] Research Center of Basic Medical Sciences, Tianjin Medical University, Tianjin 300070, China

[3] Key Laboratory of Immune Microenvironment and Disease (Ministry of Education), Tianjin Medical University, Tianjin 300070, China

[4] School of Biomedical Engineering and Technology, Tianjin Medical University, Tianjin 300070, China

[*] Correspondence: duanxc@tmu.edu.cn (X.-C.D.); yuyang@tmu.edu.cn (Y.Y.); Tel.: 86-22-83336680 (X.-C.D.); Fax: 86-22-83336560 (X.-C.D.)

[†] These authors contributed equally to this work.

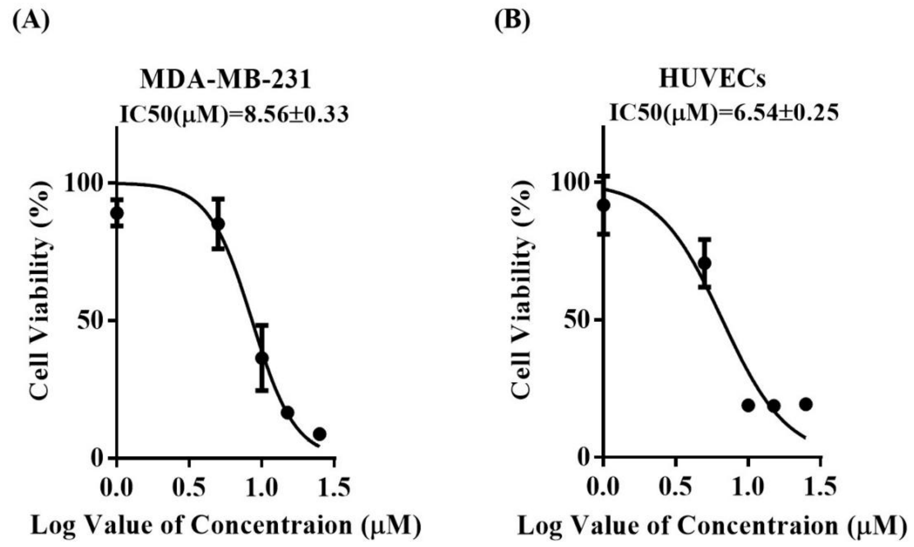

Figure S1. The cytotoxicity of compound **1** on MDA-MB-231 cells (**A**) and HUVECs (**B**).

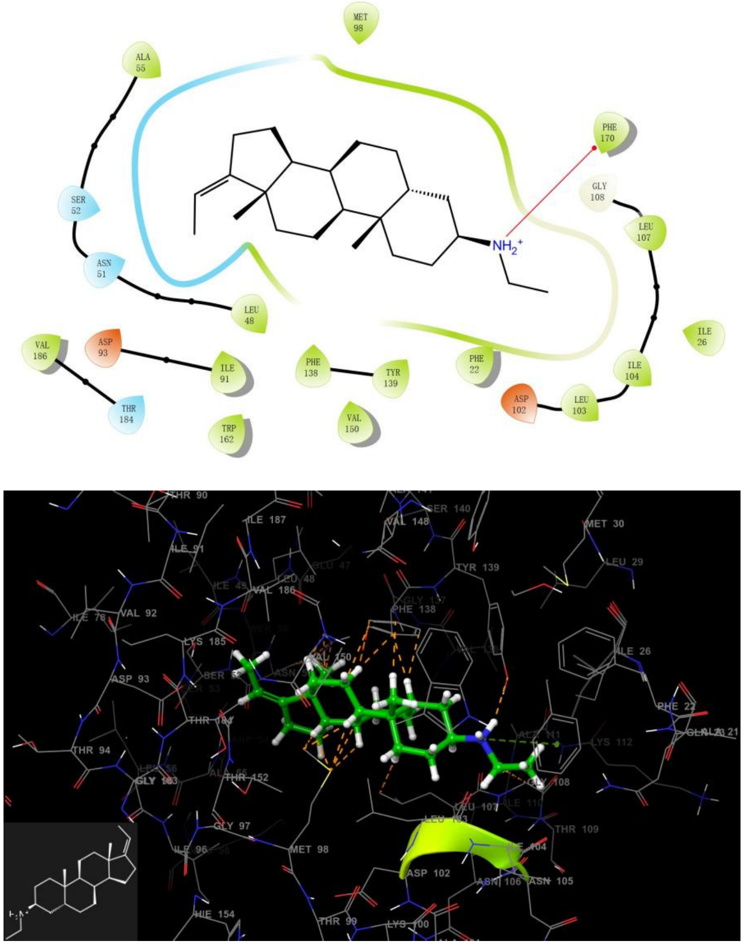

Figure S2. Detailed interaction model of compound **1** and HSP90α in the best docking pose.

Table S1. Inhibitory effects of derivatives on the migration of MDA-MB-231 cells induced by chemokine EGF. （23f represents compound **1**）

No.	IC_{50}[a] (μM)	No.	IC_{50}[a] (μM)	No.	IC_{50}[a] (μM)
**5**	0.09	**22a**	7.37	**24e**	>50
**6**	2.21	**22b**	>50	**24f**	20.15
**11**	Tox[c]	**22c**	0.03	**25a**	Tox[c]
**12**	1.08	**22d**	2.34	**25b**	Tox[c]
**17a**	0.48	**23a**	5.51	**25c**	21.39
**17b**	18.93	**23b**	4.36	**25d**	15.92
**17c**	28.54	**23c**	>50	**25e**	4.77
**18a**	0.30	**23d**	26.74	**25f**	Tox[c]
**18b**	Tox[c]	**23e**	1.21	**26a**	>50
**18c**	Tox[c]	**23f (1)**	0.17	**26b**	0.26
**21a**	2.14	**24a**	7.45	**26c**	0.85
**21b**	Tox[c]	**24b**	24.23	**26d**	Tox[c]
**21c**	>50	**24c**	2.48	**26e**	Tox[c]
**21d**	0.84	**24d**	>50	**26f**	32.84
LY294002[b]	0.38				

[a] IC_{50} represents the concentration of the compound producing 50% inhibition against human MDA-MB-231 breast cancer cells.
[b] positive control

[c] Tox represents cytotoxicity in the test concentration.
